# SMYD3–CDCP1 Axis Drives EMT and CAF Activation in Colorectal Cancer and Is Targetable for Oxaliplatin Sensitization

**DOI:** 10.3390/biomedicines13112737

**Published:** 2025-11-09

**Authors:** Liming Zhao, Zhexue Wang, Pu Cheng, Guoli Sheng, Mingyu Han, Zhaoxu Zheng

**Affiliations:** 1 Department of Colorectal Surgery, National Cancer Center/National Clinical Research Center for Cancer/Cancer Hospital, Chinese Academy of Medical Sciences and Peking Union Medical College, No. 17 South Lane of Panjiayuan, Chaoyang District, Beijing 100020, China; 2 The State Key Laboratory of Molecular Oncology, National Cancer Center/National Clinical Research Center for Cancer/Cancer Hospital, Chinese Academy of Medical Sciences and Peking Union Medical College, No. 17 South Lane of Panjiayuan, Chaoyang District, Beijing 100020, China; 3 Department of Urology, National Cancer Center/National Clinical Research Center for Cancer/Cancer Hospital, Chinese Academy of Medical Sciences and Peking Union Medical College, No. 17 South Lane of Panjiayuan, Chaoyang District, Beijing 100020, China; 4 Department of Hepatobiliary Surgery, National Cancer Center/National Clinical Research Center for Cancer/Cancer Hospital, Chinese Academy of Medical Sciences and Peking Union Medical College, No. 17 South Lane of Panjiayuan, Chaoyang District, Beijing 100020, China

**Keywords:** SMYD3, CDCP1, colorectal cancer, metastasis, CAF, oxaliplatin

## Abstract

**Background**: Colorectal cancer (CRC) mortality is predominantly driven by liver metastasis and poor responsiveness to chemotherapy. The histone methyltransferase SMYD3 has been implicated in oncogenic transcriptional programs; however, its downstream effectors and microenvironmental roles in CRC remain unclear. **Methods**: We investigated whether SMYD3 regulates the transcription and function of the membrane receptor CDCP1, which mediates Src/PKCδ signaling and promotes invasion and stromal remodeling. A combination of molecular assays, including ChIP-qPCR, Western blotting, and co-culture experiments, was employed to examine the SMYD3–CDCP1 axis and its impact on epithelial–mesenchymal transition (EMT), cancer-associated fibroblast (CAF) activation, and oxaliplatin (OXA) sensitivity. Results: SMYD3 directly bound to the CDCP1 promoter and catalyzed H3K4me3 enrichment, thereby enhancing CDCP1 transcription. Upregulated CDCP1 activated Src/PKCδ signaling, facilitating EMT and CAF activation within the tumor microenvironment. Genetic suppression of SMYD3 reduced metastatic potential and improved oxaliplatin response in vivo, while genetic or pharmacologic perturbation attenuated tumor–stroma crosstalk and enhanced oxaliplatin sensitivity in vitro. **Conclusions**: The SMYD3–CDCP1 axis drives CRC progression by epigenetically promoting CDCP1 transcription and remodeling the tumor microenvironment. Targeting this pathway may provide a promising therapeutic strategy to restrain metastasis and enhance chemotherapy efficacy in CRC.

## 1. Introduction

Colorectal cancer (CRC) is the third most commonly diagnosed malignancy worldwide and a leading cause of cancer death [1,2]. Despite improvements in screening and systemic therapy, prognosis for patients with metastatic disease—particularly with liver involvement—remains poor [3,4,5]. Tumor cell plasticity, including epithelial–mesenchymal transition (EMT), and reciprocal crosstalk with cancer-associated fibroblasts (CAFs) are recognized drivers of invasion, immune evasion and chemoresistance [6,7,8]. Defining tractable regulators that coordinately control these processes is essential for translational progress.

SMYD3 is a SET-domain lysine methyltransferase best known for depositing H3K4me3 at active promoters and for methylating selected non-histone substrates [9,10,11]. Aberrant SMYD3 expression has been reported in several solid tumors, where it supports proliferative and invasive phenotypes [12,13,14]. However, in CRC the clinical association of SMYD3 with metastatic behavior and survival, its direct transcriptional targets, and its contribution to stromal activation have not been comprehensively delineated. Moreover, whether SMYD3 activity can be therapeutically leveraged to improve the performance of standard chemotherapy is uncertain.

CDCP1 (CUB-domain containing protein 1) is a transmembrane glycoprotein that potentiates motility and survival by engaging Src family kinases and PKCδ [15,16,17]. Elevated CDCP1 correlates with aggressive disease and poor outcomes in multiple cancers, yet the upstream epigenetic mechanisms that establish and maintain CDCP1 expression in CRC are largely unexplored [18,19]. Given SMYD3’s function as a promoter-proximal methyltransferase, we hypothesized that SMYD3 may directly activate CDCP1 transcription, thereby coupling epigenetic regulation to pro-invasive signaling [20,21].

We therefore pursued an integrated clinical–mechanistic study to: (i) define SMYD3 expression patterns and prognostic significance in CRC cohorts; (ii) determine the impact of SMYD3 on cell motility, EMT, and experimental liver colonization; (iii) identify and validate CDCP1 as a downstream effector, including the chromatin mechanism by which SMYD3 regulates CDCP1 via promoter H3K4 trimethylation; (iv) evaluate how this axis modulates CAF activation through Src/PKCδ signaling; and (v) test the translational potential of pathway blockade—genetic or pharmacologic—to restrain metastasis and enhance oxaliplatin responsiveness in vivo.

Our data show that SMYD3 is upregulated in CRC and independently predicts poor survival; that SMYD3 promotes EMT, invasion and liver colonization; that CDCP1 is a direct chromatin target activated by SMYD3-dependent H3K4me3; and that CDCP1 is required for SMYD3-driven metastatic traits and for paracrine CAF activation. Importantly, inhibiting the SMYD3/CDCP1/Src axis deactivates CAFs, curbs hepatic metastatic outgrowth, and sensitizes tumors to oxaliplatin, nominating this pathway as a clinically actionable vulnerability in CRC.

## 2. Materials and Methods

### 2.1. Clinical Specimens and Tissue Microarray

Fresh tumor and matched adjacent normal mucosa from CRC patients were collected at resection, snap-frozen in liquid nitrogen for RNA and protein extraction (20 matched pairs for qPCR and 8 matched pairs for immunoblotting). Inclusion criteria were histologically confirmed primary CRC, availability of matched adjacent normal mucosa and clinicopathologic data, and adequate tissue quality for the intended assays. Exclusion criteria were receipt of neoadjuvant therapy when not explicitly analyzed, inadequate tissue or assay failure on QC, and missing clinicopathologic records. A CRC tissue microarray (TMA) containing 80 paired tumor cores and matched normals was used for IHC-based scoring of SMYD3 and CDCP1. Clinicopathologic data were abstracted from records.

### 2.2. Public Datasets and Bioinformatics Analysis

Harmonized transcript-per-million (TPM) values for SMYD3 and CDCP1 were obtained from The Cancer Genome Atlas (TCGA) and Genotype-Tissue Expression (GTEx) datasets via the UCSC Xena Toil pipeline. Unless otherwise specified, expression values were transformed as log_2_(TPM + 1). In TCGA-COAD, cases were stratified by M stage (M0 vs. M1). Time-dependent receiver operating characteristic (ROC) curves were generated using the timeROC package, and survival analyses were performed with survival and survminer (Cox proportional hazards model and Kaplan–Meier analysis with log-rank test) in R (version 4.1.1). To identify genes differentially expressed upon SMYD3 depletion, RNA-seq data from GSE67790 were processed and analyzed (|log_2_FC| ≥ 1, FDR < 0.05). Kyoto Encyclopedia of Genes and Genomes (KEGG) pathway enrichment was performed using the clusterProfiler package, with *p*-values adjusted by the Benjamini–Hochberg method.

### 2.3. Cell Lines and Culture

SW480 (ATCC CCL-228) and HCT116 (ATCC CCL-247) human colorectal cancer cell lines were obtained from the American Type Culture Collection (ATCC, Manassas, VA, USA) and maintained under standard culture conditions. SW480 cells were grown in Dulbecco’s Modified Eagle Medium (DMEM), and HCT116 cells were cultured in Roswell Park Memorial Institute (RPMI-1640) medium. Both media were supplemented with 10% fetal bovine serum (FBS) and 1% penicillin–streptomycin. All cells were incubated at 37 °C in a humidified atmosphere containing 5% CO_2_. Cell line authenticity was verified by short tandem repeat (STR) profiling, and all lines were confirmed to be mycoplasma-free by PCR testing. Cells were used for experiments within 20 passages after thawing.

### 2.4. Inhibitors and Drugs

BCI-121 (SMYD3 inhibitor; Selleck Chemicals, Houston, TX, USA) and SU6656 (Src family kinase inhibitor; MedChemExpress, Monmouth Junction, NJ, USA) were dissolved in dimethyl sulfoxide (DMSO). Unless otherwise specified, cells were treated with BCI-121 (50 or 100 µM) or SU6656 (10 µM) for 24 h, with DMSO (≤0.1%) serving as the vehicle control. Oxaliplatin (OXA) for in vivo experiments was freshly prepared according to the manufacturer’s instructions in 5% glucose solution.

### 2.5. Lentiviral Constructs and Transduction

Lentiviral short hairpin RNA (shRNA) constructs targeting SMYD3 (sh1-SMYD3, sh2-SMYD3) and CDCP1 (sh-CDCP1), along with non-targeting control (sh-NC), were cloned into the pLKO.1 lentiviral backbone (GeneChem, Shanghai, China). Overexpression constructs (LV-SMYD3 and LV-CDCP1) were generated using a CMV promoter–driven lentiviral vector encoding full-length cDNA sequences. Lentiviruses were packaged in 293T cells using psPAX2 and pMD2.G helper plasmids and used to transduce target cells at a multiplicity of infection (MOI) of 10 in the presence of 8 µg/mL polybrene (Sigma-Aldrich, St. Louis, MO, USA). Forty-eight hours after transduction, cells were selected with puromycin (2 µg/mL) for 5–7 days to establish stable lines. shRNA target sequences are listed in Supplementary Table S1.

### 2.6. Quantitative Real-Time PCR (qPCR)

Total RNA was extracted using TRIzol reagent (Invitrogen, Carlsbad, CA, USA). Complementary DNA (cDNA) was synthesized from 1 µg of total RNA using random hexamers and a reverse transcription kit (PrimeScript RT reagent kit, Takara, Shiga, Japan). Quantitative PCR was performed using SYBR Green Master Mix (Applied Biosystems, Foster City, CA, USA) on an ABI 7500 Real-Time PCR System. GAPDH was used as the endogenous reference gene, and relative mRNA expression levels were calculated using the 2^–ΔΔCt^ method. Primer sequences are provided in Supplementary Table S2.

### 2.7. Western Blotting

Cells or tissues were lysed in RIPA buffer (Beyotime, Shanghai, China) containing protease and phosphatase inhibitor cocktails (Roche, Basel, Switzerland). Protein concentrations were determined using the BCA Protein Assay Kit (Thermo Fisher Scientific, Waltham, MA, USA), and equal amounts (20–40 µg) of protein were separated by SDS-PAGE and transferred to PVDF membranes (Millipore, Billerica, MA, USA) using a semi-dry transfer system. Membranes were blocked with 5% non-fat milk in TBST for 1 h at room temperature, incubated overnight at 4 °C with primary antibodies, and then incubated with HRP-conjugated secondary antibodies (Jackson ImmunoResearch, West Grove, PA, USA) for 1 h at room temperature. Protein bands were visualized using enhanced chemiluminescence (ECL) detection reagent (Thermo Fisher Scientific) and quantified using ImageJ software (version 1.54, NIH, Bethesda, MD, USA). Antibodies included SMYD3, CDCP1, H3K4me3, E-cadherin, ZEB1, Snail, p-Src (Y416), Src, p-PKCδ (Y311), PKCδ, α-SMA, FAP, PDGFRβ, S100A4, and GAPDH; detailed information on antibody vendors and catalog numbers is provided in Supplementary Table S3.

### 2.8. Transwell Migration and Matrigel Invasion

Transwell chambers with 8-µm pore polycarbonate membranes (Corning, Corning, NY, USA) were used for migration and invasion assays. For invasion assays, the upper surface of the insert was pre-coated with Matrigel (Corning, 1:8 dilution in serum-free medium). Cells were serum-starved in serum-free DMEM (or RPMI-1640) for 8 h before seeding. A total of 5 × 10^4^ to 1 × 10^5^ cells suspended in 200 µL of serum-free medium were added to the upper chamber, and 600 µL of medium containing 10% FBS was added to the lower chamber as a chemoattractant. After incubation for 24 h (migration) or 36 h (invasion), non-migrated cells on the upper membrane were removed with a cotton swab. The migrated or invaded cells on the lower surface were fixed with methanol for 10 min, stained with 0.1% crystal violet for 15 min, and rinsed with PBS. The number of stained cells was counted in five randomly selected fields per insert under a light microscope in a blinded manner.

### 2.9. Co-Culture with Cancer-Associated Fibroblasts (CAFs)

Primary CAFs derived from CRC tissues were used for co-culture experiments. Indirect co-culture was performed using 0.4-µm pore polycarbonate Transwell inserts (Corning, Corning, NY, USA), with CAFs seeded in the lower chamber and CRC cells in the upper chamber at an approximately 1:1 ratio, and co-cultured for 48 h. Where indicated, BCI-121 or SU6656 was added to the tumor-cell compartment at the beginning of co-culture. CAF activation was evaluated by qPCR and immunoblotting for α-SMA, FAP, PDGFRβ, and S100A4 expression.

### 2.10. Chromatin Immunoprecipitation (ChIP)

Approximately 1 × 10^6^ CRC cells were cross-linked in 1% formaldehyde in PBS for 10 min at room temperature and quenched with 125 mM glycine for 5 min. Nuclei were isolated, and chromatin was sonicated to 200–500 bp fragments using a Bioruptor sonicator (Diagenode, Denville, NJ, USA); shearing efficiency was confirmed by agarose gel electrophoresis. Ten percent of the sheared chromatin was reserved as input control, and the remainder was incubated overnight at 4 °C with ChIP-grade antibodies against SMYD3 or H3K4me3, or with normal IgG (antibody details in Supplementary Table S3), followed by incubation with Protein A/G magnetic beads (2 h, 4 °C). Immune complexes were washed sequentially with low-salt, high-salt, LiCl, and TE buffers, and DNA was eluted and reverse cross-linked at 65 °C overnight. Samples were then treated with RNase A and Proteinase K, and DNA was purified using a PCR purification kit (Qiagen, Hilden, Germany). Two primer pairs (P1 and P2) within the proximal CDCP1 promoter region (~−1.5 to −0.0 kb from the transcription start site, GRCh38) were used for amplification. Enrichment was quantified as a percentage of input using the formula: %Input = 100 × 2^(Ct_adjInput − Ct_IP), where Ct_adjInput = Ct_input − 3.32. End-point PCR products (100–150 bp) corresponding to P1 and P2 regions were visualized on 2% agarose gels stained with GelRed.

### 2.11. Immunohistochemistry and H-Score

4 µm paraffin-embedded tissue sections were deparaffinized and rehydrated. Antigen retrieval was performed in citrate buffer (pH 6.0) using a pressure cooker, followed by quenching of endogenous peroxidase activity with 3% hydrogen peroxide and blocking with 5% bovine serum albumin (BSA) for 30 min at room temperature. Slides were incubated overnight at 4 °C with primary antibodies against SMYD3, CDCP1, α-SMA, and FAP (antibody details in Supplementary Table S3), followed by incubation with HRP-conjugated polymer secondary antibodies (DAKO, Glostrup, Denmark) for 30–60 min at room temperature. Immunoreactivity was visualized using 3,3′-diaminobenzidine (DAB) substrate, and nuclei were counterstained with hematoxylin. Two independent pathologists, blinded to clinical data and group allocation, evaluated staining intensity and extent. The H-score (range, 0–300) was calculated as the product of staining intensity (0–3) and the percentage of positive cells (0–100%). Cases were stratified into “low” and “high” expression groups based on the optimal cut-off derived from receiver operating characteristic (ROC) curve analysis.

### 2.12. Experimental Liver Colonization and Treatments

Six-week-old male BALB/c nude mice (*n* = 5 per group) were randomized prior to treatment. Luciferase-labeled CRC cells (1 × 10^6^ cells in 50 µL PBS) were injected into the spleen under anesthesia, followed by splenectomy to prevent local tumor growth. Experimental groups included vehicle control, SMYD3-overexpressing (LV-SMYD3), CDCP1-knockdown (sh-CDCP1), and combination (LV-SMYD3 + sh-CDCP1) cohorts, as indicated. For chemotherapy, oxaliplatin (5 mg/kg in 5% glucose) was administered intraperitoneally twice weekly for 3 weeks.

In vivo bioluminescence imaging was performed using an IVIS Spectrum system (PerkinElmer, Waltham, MA, USA) 10 min after intraperitoneal injection of D-luciferin (150 mg/kg). Hepatic photon flux was quantified within a fixed region of interest (ROI) using Living Image software (version 4.7.3, PerkinElmer, Waltham, MA, USA). At the experimental endpoint, livers were excised, weighed, and fixed in 10% neutral-buffered formalin for hematoxylin–eosin (H&E) and immunohistochemical (IHC) analysis. Metastatic burden was quantified as the percentage of tumor area relative to total liver area on whole-slide digital scans. Survival cohorts were monitored until humane endpoints. Randomization was performed by an independent laboratory member, and histologic quantification was conducted in a blinded manner.

### 2.13. Statistical Analysis

Data are presented as mean ± SEM from at least three independent experiments. Statistical analyses were performed using GraphPad Prism (version 10.1; GraphPad Software, San Diego, CA, USA) or R (version 4.1.1). Comparisons between two groups were conducted using two-tailed unpaired Student’s *t*-tests, whereas comparisons among multiple groups were analyzed by one-way ANOVA. Pearson correlation analysis was used to evaluate associations between gene expression levels. Survival curves were generated by the Kaplan–Meier method and compared using the log-rank test. Hazard ratios (HRs) and 95% confidence intervals (CIs) were calculated by Cox proportional hazards regression. A *p*-value < 0.05 was considered statistically significant.

## 3. Results

### 3.1. SMYD3 Is Upregulated in Colorectal Cancer and Associates with Metastasis and Poor Prognosis

**Figure 1:**
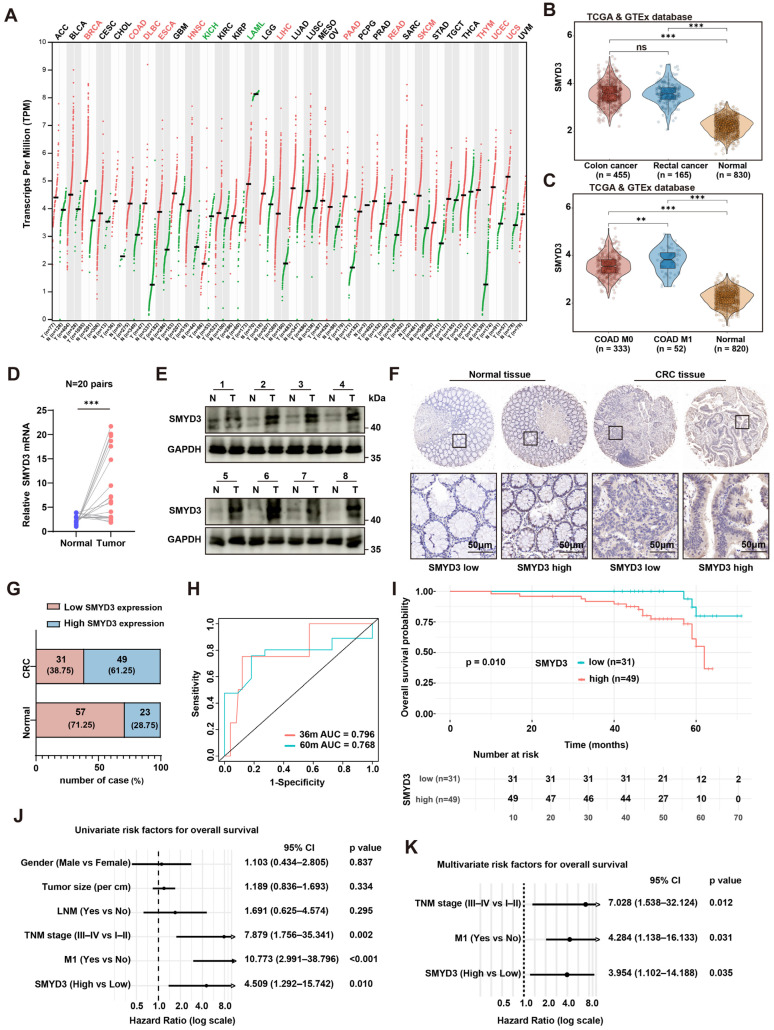
SMYD3 is upregulated in colorectal cancer and associates with metastasis and poor prognosis. (**A**) Pan-cancer landscape of SMYD3 mRNA (log2 [TPM+1]) across TCGA/GTEx cohorts; red dots indicate tumor, green dots indicate normal. (**B**) Violin plots of SMYD3 expression in colon cancer (n = 455), rectal cancer (n = 165) and normal colorectal tissues (n = 830) from TCGA/GTEx. *** *p* < 0.001; ns, not significant. (**C**) SMYD3 levels in TCGA-COAD stratified by metastatic status (M0, n = 333; M1, n = 52) versus normal (n = 820). ** *p* < 0.01, *** *p* < 0.001. (**D**) Relative SMYD3 mRNA in 20 paired tumors (T) and adjacent normals (N) by qPCR; lines connect pairs (*** *p* < 0.001). (**E**) Immunoblotting of SMYD3 in 8 matched pairs (N/T); GAPDH loading control; cases 1–4 (upper) and 5–8 (lower). (**F**) Representative immunohistochemistry (IHC) for SMYD3 in normal colorectal mucosa and CRC tissues, illustrating low and high expression. Black squares mark regions shown at higher magnification below. Scale bars: 50 μm. (**G**) Distribution of SMYD3 IHC categories in normals and CRCs: SMYD3-low vs. SMYD3-high with counts and percentages. (**H**) Time-dependent ROC curves of SMYD3 for 36- and 60-month overall survival (AUC = 0.796 and 0.768, respectively). (**I**) Kaplan–Meier overall survival according to SMYD3 IHC category (low, n = 31; high, n = 49); *p* = 0.010 by log-rank test. (**J**) Univariable Cox regression forest plot for OS including gender, tumor size, lymph-node metastasis (LNM), TNM stage, M1 status and SMYD3; hazard ratios (HRs) with 95% CIs and *p* values shown. (**K**) Multivariable Cox regression demonstrating TNM stage, M1 status and SMYD3-high as independent prognostic factors (HRs with 95% CIs and *p* values).

Pan-cancer expression analysis using GEPIA2, which integrates RNA-seq data from TCGA and GTEx, revealed that SMYD3 mRNA is widely expressed across solid tumors and notably upregulated in several malignancies, including colorectal cancer (CRC) (Figure 1A). Consistent with this finding, analysis of the TCGA/GTEx CRC cohort demonstrated that SMYD3 expression was significantly higher in colon (*n* = 455) and rectal cancers (*n* = 165) than in normal colorectal tissues (*n* = 830), with no significant difference between colon and rectal subtypes (Figure 1B). Further stratification of TCGA-COAD cases showed that tumors with distant metastasis (M1, *n* = 52) exhibited markedly higher SMYD3 expression than both non-metastatic tumors (M0, *n* = 333) and normal controls (*n* = 820) (Figure 1C).

These observations were validated in our clinical samples. In 20 paired CRC and adjacent tissues, SMYD3 mRNA was consistently increased in tumors (paired qPCR, *p* < 0.001; Figure 1D), and SMYD3 protein was elevated in the majority of tumors by immunoblotting of 8 matched pairs (Figure 1E). Immunohistochemistry (IHC) confirmed stronger nuclear SMYD3 staining in CRC compared with normal mucosa (Figure 1F). Based on IHC scores, 61.25% of CRCs were classified as SMYD3-high vs. 28.75% of normals, whereas 38.75% of CRCs and 71.25% of normals were SMYD3-low (Figure 1G).

Time-dependent ROC analysis indicated good prognostic discrimination of SMYD3 for overall survival (OS) with 36- and 60-month AUCs of 0.796 and 0.768, respectively (Figure 1H). Kaplan–Meier analysis showed that patients with SMYD3-high tumors had significantly shorter OS than those with SMYD3-low tumors (*p* = 0.010; Figure 1I). In univariable Cox models, advanced TNM stage (III–IV vs. I–II; HR = 7.879, 95% CI 1.756–35.341, *p* = 0.002), presence of distant metastasis (M1 vs. M0; HR = 10.773, 95% CI 2.991–38.796, *p* < 0.001), and high SMYD3 (HR = 4.509, 95% CI 1.292–15.742, *p* = 0.010) were risk factors for death (Figure 1J). In multivariable analysis, SMYD3-high remained an independent predictor of worse OS (HR = 3.954, 95% CI 1.102–14.188, *p* = 0.035), together with TNM stage (HR = 7.028, 95% CI 1.538–32.124, *p* = 0.012) and M1 status (HR = 4.284, 95% CI 1.138–16.133, *p* = 0.031) (Figure 1K). Collectively, SMYD3 is upregulated at both transcript and protein levels in CRC, associates with metastatic disease, and independently predicts poor survival.

### 3.2. SMYD3 Drives Colorectal Cancer Cell Motility, EMT, and Liver Metastasis

**Figure 2:**
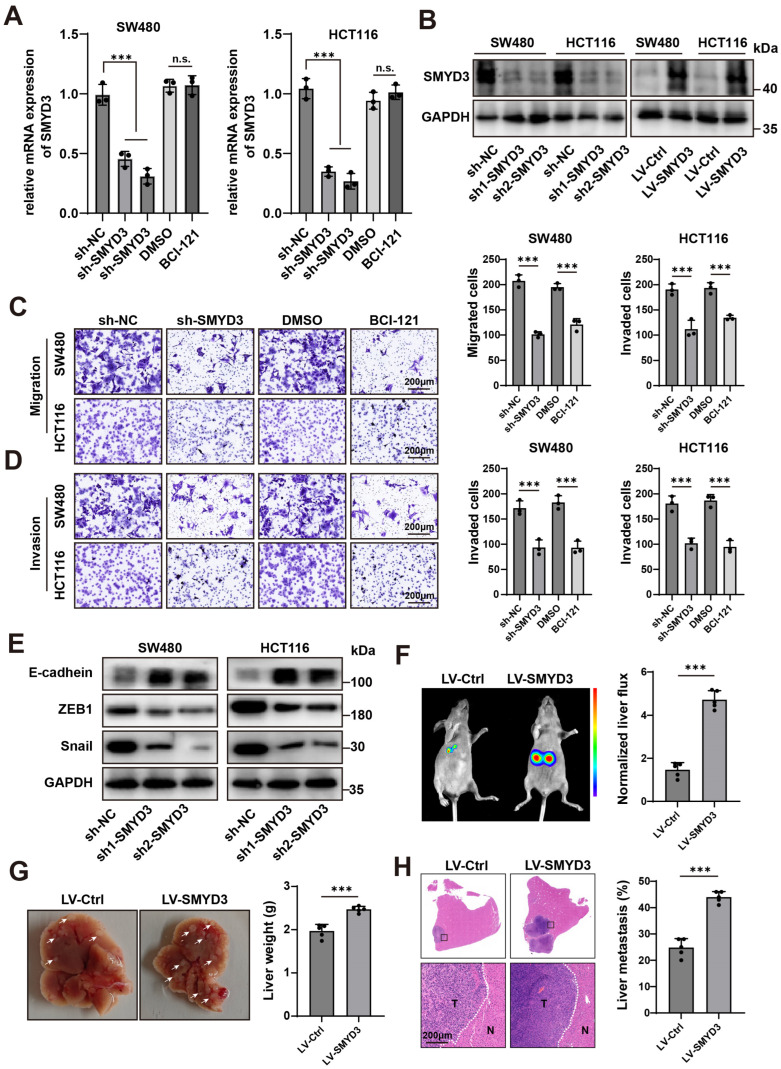
SMYD3 drives colorectal cancer cell motility, EMT, and liver metastasis. (**A**) qPCR showing relative SMYD3 mRNA in SW480 and HCT116 cells transduced with control shRNA (sh-NC) or two independent shRNAs (sh1-SMYD3, sh2-SMYD3). Cells treated with DMSO or the SMYD3 inhibitor BCI-121 are shown for comparison. *** *p* < 0.001; n.s., not significant. (**B**) Immunoblot verification of SMYD3 knockdown and lentiviral overexpression (LV-SMYD3); GAPDH, loading control. (**C**) Transwell migration assay after SMYD3 inhibition. **Left**: Representative crystal-violet–stained images of migrated SW480 and HCT116 cells in uncoated inserts under sh-NC, sh-SMYD3, DMSO, and BCI-121. **Right**: Quantification of migrated cells for each condition. Bars show mean ± SEM from ≥3 independent experiments; one-way ANOVA with post hoc multiple comparisons versus the corresponding control (sh-NC or DMSO); *** *p* < 0.001. Scale bars, 200 μm. (**D**) Matrigel invasion assay after SMYD3 inhibition. **Left**: Representative images of invaded SW480 and HCT116 cells in Matrigel-coated inserts under sh-NC, sh-SMYD3, DMSO, and BCI-121. **Right**: Quantification of invaded cells. *** *p* < 0.001. Scale bars, 200 μm. (**E**) Western blot analysis of epithelial–mesenchymal transition (EMT) markers after SMYD3 knockdown in SW480 and HCT116 cells. Two independent shRNAs (sh1-SMYD3, sh2-SMYD3) increase E-cadherin and reduce ZEB1 and Snail compared with the sh-NC. GAPDH serves as the loading control. (**F**) In vivo bioluminescence imaging (BLI) in an experimental liver-colonization model comparing LV-Ctrl and LV-SMYD3 groups. **Left**, representative BLI images; **right**, quantification of normalized liver ROI photon flux. Dots denote individual mice (n = 5 per group); bars = mean ± SEM; two-tailed Student’s *t* test, *** *p* < 0.001. (**G**) Representative gross livers and corresponding liver weights from the mice in (**F**); mean ± SEM, *** *p* < 0.001. (**H**) H&E staining of liver sections showing metastatic nodules (T) adjacent to normal parenchyma (N). **Right**, quantification of the metastatic area (%); *** *p* < 0.001. Scale bars, 200 μm.

To define the functional contribution of SMYD3 to colorectal cancer (CRC) progression, we combined genetic loss-of-function, pharmacologic inhibition, and gain-of-function approaches in SW480 and HCT116 cells. Two independent shRNAs efficiently suppressed SMYD3 transcripts as measured by qPCR, whereas the small-molecule SMYD3 inhibitor BCI-121 did not alter SMYD3 mRNA relative to vehicle (Figure 2A). Immunoblotting confirmed robust depletion of SMYD3 protein by both shRNAs and successful overexpression following lentiviral transduction (LV-SMYD3) (Figure 2B), establishing reagents for downstream phenotypic testing.

Functionally, SMYD3 loss reduced cancer cell motility. In Transwell assays, SMYD3 knockdown significantly decreased the number of migrated and invaded cells in both lines, and acute enzymatic inhibition by BCI-121 phenocopied the genetic suppression (Figure 2C,D). These concordant results across two models indicate that SMYD3 enzymatic activity is required for efficient migration and extracellular-matrix invasion.

Given the close link between motility and epithelial–mesenchymal transition (EMT), we next examined canonical EMT markers. Silencing SMYD3 led to a molecular shift consistent with EMT reversal: E-cadherin was upregulated, while ZEB1 and Snail were downregulated in both cell lines (Figure 2E). Thus, SMYD3 sustains an EMT-like program that favors a migratory and invasive phenotype.

We then tested whether SMYD3 enhances metastatic competence in vivo. In an intrasplenic injection model of experimental liver metastasis, mice injected with LV-SMYD3 cells exhibited markedly higher hepatic bioluminescent signals than LV-Ctrl animals (*p* < 0.001; Figure 2F). Consistently, gross examination revealed increased liver weights and a greater number of metastatic nodules in the LV-SMYD3 group, which was corroborated by H&E staining showing tumor foci (T) infiltrating adjacent normal parenchyma (N) (*p* < 0.001; Figure 2G,H).

Collectively, these data demonstrate that SMYD3 is a key promigratory and proinvasive driver in CRC that promotes EMT and confers a strong advantage for liver colonization in vivo.

### 3.3. SMYD3 Upregulates CDCP1 and Their Co-Expression Predicts Adverse Prognosis in Colorectal Cancer

**Figure 3:**
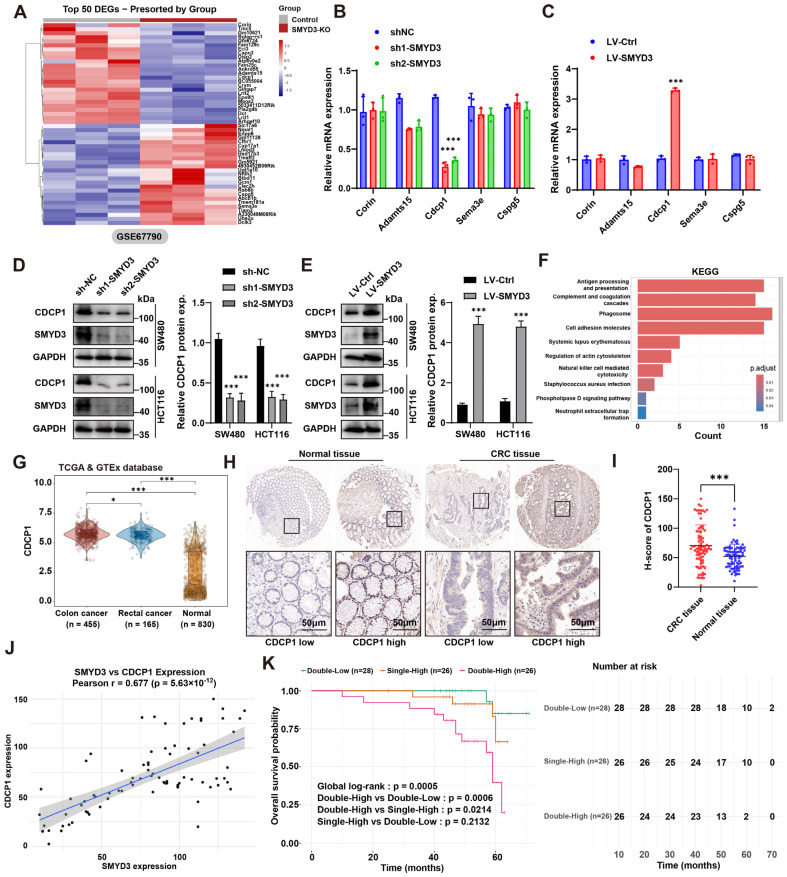
SMYD3 upregulates CDCP1 and their co-expression predicts adverse prognosis in colorectal cancer. (**A**) Heatmap of the top 50 differentially expressed genes between SMYD3-KO and control cells from GSE67790, showing a distinct SMYD3-dependent signature. (**B**) qPCR validation of candidate transcripts in SW480 and HCT116 after SMYD3 knockdown (sh1-SMYD3, sh2-SMYD3) versus sh-NC. CDCP1 is strongly reduced; other candidates show minimal change. *** *p* < 0.001. (**C**) qPCR showing increased CDCP1 mRNA following lentiviral SMYD3 overexpression relative to LV-Ctrl. *** *p* < 0.001. (**D**) Immunoblot of CDCP1 and SMYD3 after knockdown in SW480 and HCT116; GAPDH, loading control. **Right**, densitometric quantification of CDCP1 protein (mean ± SEM); *** *p* < 0.001. (**E**) Immunoblot verifying upregulation of CDCP1 protein upon SMYD3 overexpression; quantification shown on the **right**; *** *p* < 0.001. (**F**) KEGG enrichment analysis of SMYD3-regulated genes highlighting pathways related to immune interaction and cell movement (e.g., antigen presentation, phagosome, ECM–receptor interaction, cell-adhesion molecules, regulation of actin cytoskeleton). Bar color indicates adjusted *p*. (**G**) Violin plots of CDCP1 expression in TCGA/GTEx cohorts: colon cancer (n = 455) and rectal cancer (n = 165) versus normal colorectal tissues (n = 830). * *p* < 0.05, *** *p* < 0.001. (**H**) Representative IHC for CDCP1 in normal colorectal mucosa and CRC tissues, illustrating low and high expression. Black squares mark regions shown at higher magnification below. Scale bars: 50 μm. (**I**) H-score comparison showing higher CDCP1 in CRC versus normal tissues, *** *p* < 0.001. (**J**) Positive correlation between SMYD3 and CDCP1 expression across CRC samples; Pearson r = 0.677, *p* = 5.63 × 10^−12^. (**K**) Kaplan–Meier overall survival by combined SMYD3/CDCP1 expression: Patients were stratified into three groups according to SMYD3 and CDCP1 expression: Double-Low (n = 28), Single-High (n = 26), and Double-High (n = 26). The overall survival was significantly different among the groups (global log-rank *p* = 0.0005). Pairwise comparisons indicated that the Double-High group had markedly poorer survival than the Double-Low (*p* = 0.0006) and Single-High (*p* = 0.0214) groups, whereas there was no significant difference between Single-High and Double-Low (*p* = 0.2132). Numbers at risk are shown below the survival curves.

To identify downstream effectors mediating the prometastatic role of SMYD3, we mined an RNA-seq dataset of SMYD3 knockout versus control cells (GSE67790). Unsupervised clustering of the top differentially expressed genes revealed a distinct SMYD3-dependent signature (Figure 3A). Among candidates linked to adhesion and motility, CDCP1 emerged as one of the most consistently downregulated transcripts upon SMYD3 loss.

We next validated these findings in CRC models. In SW480 and HCT116 cells, SMYD3 silencing with two independent shRNAs markedly reduced CDCP1 mRNA, whereas other screened candidates showed minimal or variable changes (Figure 3B). Conversely, lentiviral SMYD3 overexpression significantly increased CDCP1 mRNA (Figure 3C). At the protein level, immunoblotting confirmed that genetic SMYD3 depletion lowered CDCP1 abundance, while SMYD3 overexpression elevated CDCP1 (densitometric quantification, *p* < 0.001; Figure 3D,E). Pathway enrichment of the SMYD3-regulated transcriptome highlighted terms tightly connected to invasion and microenvironmental interactions—including antigen processing and presentation, complement and coagulation cascades, phagosome, cell-adhesion molecules, ECM–receptor interaction, and regulation of actin cytoskeleton—supporting a functional link between SMYD3 activity and pro-metastatic programs (Figure 3F).

Interrogation of TCGA/GTEx cohorts demonstrated that CDCP1 expression was significantly higher in colon (n = 455) and rectal cancers (n = 165) compared with normal colorectal tissues (n = 830) (Figure 3G). Immunohistochemistry on a CRC tissue microarray further validated this observation: tumor epithelium exhibited stronger membrane CDCP1 staining than adjacent normal mucosa, and H-scores were markedly increased in tumors (*p* < 0.001; Figure 3H,I). Importantly, CDCP1 levels positively correlated with SMYD3 expression across patient samples (Pearson r = 0.677, *p* = 5.63 × 10^−12^), indicating a tight association in clinical tissues (Figure 3J).

We then evaluated the prognostic value of the SMYD3–CDCP1 axis. Patients were stratified by combined expression into Double-Low (both low), Single-High (either high), and Double-High (both high). Kaplan–Meier analysis revealed significant separation of overall survival curves (global log-rank *p* = 0.0005): the Double-High group displayed the worst survival, significantly inferior to Double-Low (*p* = 0.0006) and Single-High (*p* = 0.0214), whereas Single-High versus Double-Low was not statistically different (*p* = 0.2132) (Figure 3K). Together, these data demonstrate that SMYD3 upregulates CDCP1 at transcript and protein levels, and that concurrent elevation of SMYD3 and CDCP1 identifies CRC patients with the poorest outcomes.

### 3.4. SMYD3 Activates CDCP1 Transcription Through Promoter H3K4 Trimethylation

**Figure 4:**
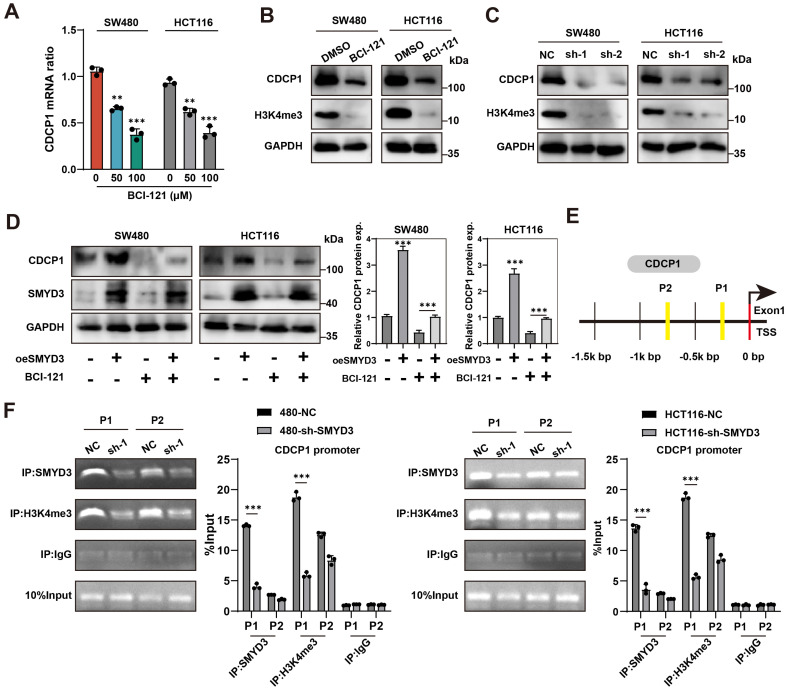
SMYD3 catalyzes H3K4 trimethylation at the CDCP1 promoter to activate CDCP1 expression. (**A**) qPCR analysis showing dose-dependent reduction in CDCP1 mRNA in SW480 and HCT116 cells treated with the SMYD3 inhibitor BCI-121 (0, 50, 100 μM). ** *p* < 0.01, *** *p* < 0.001. (**B**) Immunoblots of CDCP1 and H3K4me3 after DMSO or BCI-121; GAPDH, loading control. (**C**) Immunoblots showing reduced CDCP1 and H3K4me3 following SMYD3 knockdown with two shRNAs (sh-1, sh-2) in SW480 and HCT116. (**D**) Western blot analysis of CDCP1 and SMYD3 expression in SW480 and HCT116 cells following SMYD3 overexpression with or without the SMYD3 inhibitor BCI-121. GAPDH served as a loading control. **Right**: densitometric quantification of CDCP1 protein levels (mean ± SEM, n ≥ 3); *** *p* < 0.001 by one-way ANOVA with post hoc comparisons. (**E**) Schematic of the CDCP1 gene and promoter region indicating two ChIP amplicons (P1, P2) located upstream of the transcription start site (TSS). (**F**) ChIP-PCR demonstrating enrichment of SMYD3 and H3K4me3 at P1 and P2 in control cells (NC) and loss of occupancy and marks following SMYD3 knockdown. IgG, negative control; “10% input”, loading control. *** *p* < 0.001 vs. NC by two-tailed *t* test.

We first examined whether enzymatic inhibition of SMYD3 affects CDCP1 expression. Exposure of SW480 and HCT116 cells to the selective SMYD3 inhibitor BCI-121 led to a dose-dependent reduction in CDCP1 mRNA (50–100 μM), with significant decreases relative to vehicle in both cell lines (*p* < 0.01–0.001; Figure 4A). Consistently, CDCP1 protein abundance fell upon BCI-121 treatment, accompanied by a global decline in H3K4me3, whereas GAPDH remained unchanged (Figure 4B). Genetic suppression of SMYD3 using two independent shRNAs phenocopied the pharmacologic inhibition: both CDCP1 protein and H3K4me3 levels were lowered in SW480 and HCT116 (Figure 4C).

To determine whether SMYD3 catalytic activity is required for CDCP1 upregulation, we performed an epistasis experiment. Lentiviral SMYD3 overexpression markedly increased CDCP1 protein in both models; co-treatment with BCI-121 attenuated this induction, returning CDCP1 to near-baseline levels (one-way ANOVA, *p* < 0.001; Figure 4D). These data indicate that the methyltransferase activity of SMYD3 is necessary for its positive regulation of CDCP1.

We next asked whether SMYD3 directly engages the CDCP1 locus. Two amplicons (P1 and P2) were designed within the proximal promoter (within ~1.5 kb upstream of the transcription start site; Figure 4E). ChIP–PCR demonstrated robust SMYD3 occupancy and enrichment of H3K4me3 at both regions in control cells, with stronger signals at P1. Importantly, SMYD3 knockdown markedly reduced SMYD3 binding and the associated H3K4me3 enrichment at P1, whereas IgG controls were negligible (two-tailed Student’s *t* test, *p* < 0.001; Figure 4F).

Taken together, the genetic (knockdown/overexpression), pharmacologic (BCI-121), and chromatin (ChIP) evidence demonstrate that CDCP1 is a direct chromatin target of SMYD3 in colorectal cancer cells. SMYD3 binds the CDCP1 promoter and catalyzes promoter H3K4 trimethylation, a modification that is required for efficient transcriptional activation of CDCP1.

### 3.5. CDCP1 Is a Functional Effector of SMYD3 That Augments Motility, Activates Protumor Fibroblasts, and Is Required for Liver Colonization

**Figure 5:**
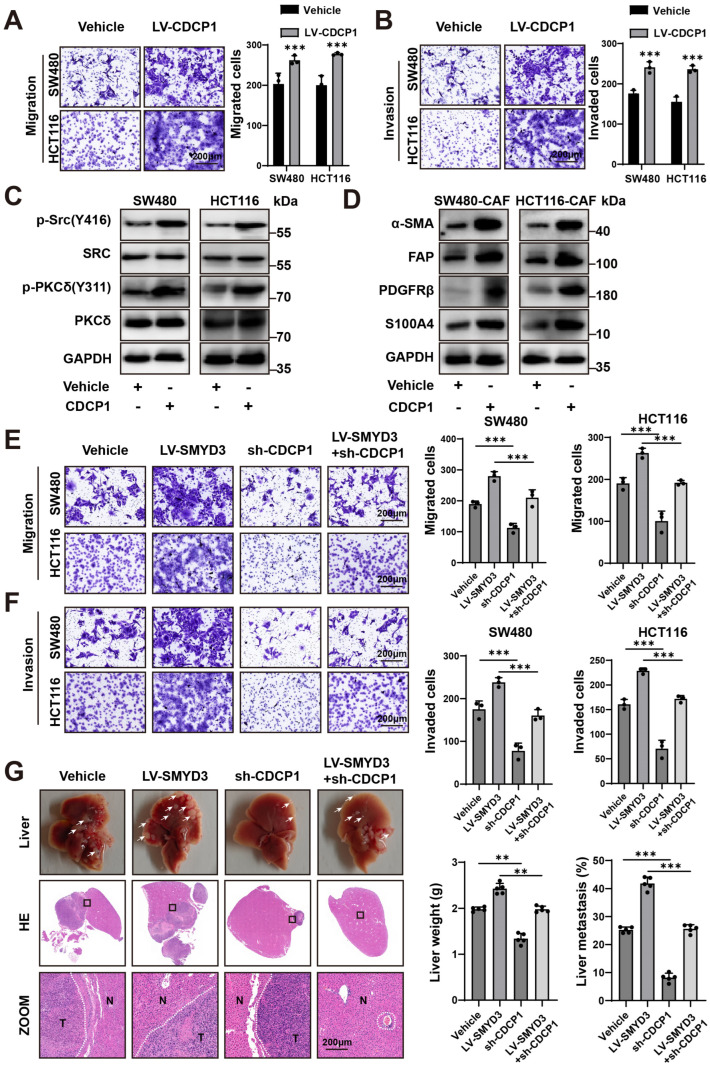
CDCP1 is a functional effector of SMYD3 that augments motility, activates protumor fibroblasts, and is required for liver colonization. (**A**) Representative Transwell migration images (**left**) and quantification (**right**) for SW480 and HCT116 transduced with vehicle or LV-CDCP1. *** *p* < 0.001. Scale bars, 200 μm. (**B**) Representative Matrigel invasion images (**left**) and quantification (**right**) under the same conditions as in (**A**). *** *p* < 0.001. Scale bars, 200 μm. (**C**) Immunoblots showing increased phosphorylation of Src (Y416) and PKCδ (Y311) upon CDCP1 overexpression; total SRC/PKCδ and GAPDH serve as controls. (**D**) Immunoblots of CAFs co-cultured with SW480 or HCT116 cells ± CDCP1 overexpression, demonstrating elevated α-SMA, FAP, PDGFRβ, and S100A4 in the CDCP1-high condition. (**E**,**F**) Transwell assays in SW480 and HCT116: migration (**E**) and invasion (**F**) for vehicle, LV-SMYD3, sh-CDCP1, and LV-SMYD3 + sh-CDCP1 groups. SMYD3 overexpression increases motility, which is blunted by CDCP1 silencing. Mean ± SEM (n ≥ 3); one-way ANOVA with post hoc testing, *** *p* < 0.001. Scale bars, 200 μm. (**G**) In vivo liver metastasis model comparing vehicle, LV-SMYD3, sh-CDCP1, and LV-SMYD3 + sh-CDCP1. **Top**, gross livers; **middle**, H&E sections with boxed regions magnified below (T, tumor; N, normal). **Right**, quantification of liver weight and metastatic area (%). ** *p* < 0.01, *** *p* < 0.001. Scale bars, 200 μm.

We asked whether CDCP1 itself is sufficient to enhance malignant traits in colorectal cancer cells. Lentiviral overexpression of CDCP1 significantly increased Transwell migration and Matrigel invasion in SW480 and HCT116 cells compared with vehicle controls (*p* < 0.001 for all comparisons; Figure 5A,B). Mechanistically, CDCP1 overexpression was accompanied by activation of canonical downstream kinases: phosphorylation of Src (Y416) and PKCδ (Y311) increased without changes in total SRC/PKCδ abundance (Figure 5C), consistent with signaling engagement by CDCP1.

Because tumor–stroma crosstalk is a major determinant of CRC metastasis, we evaluated fibroblast activation in a cancer cell–CAF co-culture system. Conditioned by CDCP1-high SW480 or HCT116 cells, CAFs displayed a protumor phenotype with upregulation of α-SMA, FAP, PDGFRβ, and S100A4 (Figure 5D), indicating that cancer-cell CDCP1 promotes fibroblast activation.

To test whether CDCP1 mediates the prometastatic program driven by SMYD3, we performed genetic epistasis. SMYD3 overexpression robustly enhanced SW480 and HCT116 migration and invasion, whereas CDCP1 knockdown significantly suppressed motility. Importantly, co-perturbation (LV-SMYD3 + sh-CDCP1) largely abrogated the migratory and invasive advantage conferred by SMYD3 (*p* < 0.001; Figure 5E,F), placing CDCP1 downstream of SMYD3.

We next examined metastatic competence in vivo using an experimental liver colonization model. Relative to vehicle controls, LV-SMYD3 markedly increased metastatic burden, reflected by higher liver weights and a greater fraction of liver occupied by tumor on H&E sections. In contrast, sh-CDCP1 reduced metastasis, and LV-SMYD3 + sh-CDCP1 significantly attenuated the SMYD3-induced increase in liver weight and metastatic area (Figure 5G). Together, these data identify CDCP1 as a key effector and requirement of SMYD3-driven invasion–metastasis programs and as a modulator of CAF activation in the CRC microenvironment.

### 3.6. Therapeutic Blockade of the SMYD3/CDCP1 Axis Deactivates CAFs, Curbs Metastatic Outgrowth, and Potentiates Oxaliplatin Efficacy

**Figure 6:**
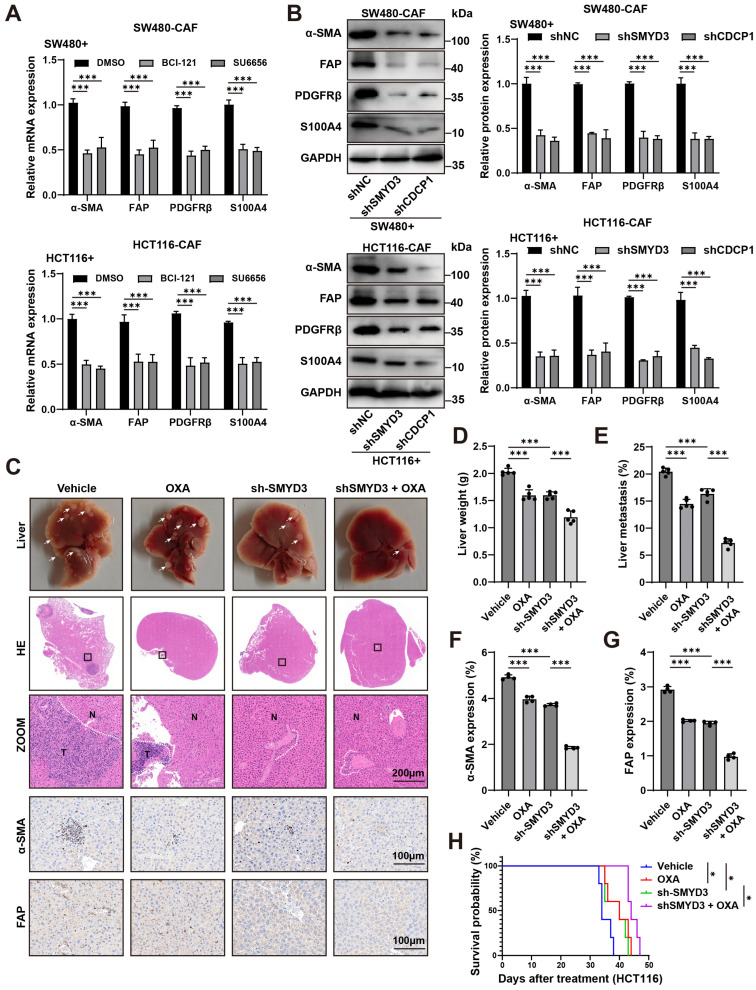
Blocking the SMYD3/CDCP1 axis deactivates CAFs and improves oxaliplatin response in colorectal cancer. (**A**) qPCR of CAF activation genes (α-SMA, FAP, PDGFRβ, S100A4) in CAFs co-cultured with SW480 (**top**) or HCT116 (**bottom**) cells and treated with DMSO, the SMYD3 inhibitor BCI-121, or the Src inhibitor SU6656. Mean ± SEM (n ≥ 3); one-way ANOVA with post hoc tests; *** *p* < 0.001. (**B**) Immunoblots of CAFs conditioned by tumor cells expressing control shRNA (shNC), shSMYD3, or shCDCP1. **Right**, densitometric quantification normalized to GAPDH (mean ± SEM, n ≥ 3); *** *p* < 0.001. (**C**) Representative livers, whole-section H&E, and high-power views from mice bearing HCT116 liver colonization treated with Vehicle, OXA, sh-SMYD3, or sh-SMYD3 + OXA (T, tumor; N, normal). White arrows mark metastatic nodules. Scale bars, 200 μm. (**D**,**E**) Quantification of liver weight and metastatic area (%) for the groups in (**C**). *** *p* < 0.001 by one-way ANOVA with post hoc testing. (**F**,**G**) IHC quantification showing reduced stromal α-SMA and FAP expression within metastases after sh-SMYD3 and further reduction with sh-SMYD3 + OXA. *** *p* < 0.001. Scale bars, 200 μm. (**H**) Kaplan–Meier survival for the four treatment arms; log-rank *p* values are indicated. * *p* < 0.05.

We first tested whether pharmacologic inhibition of the SMYD3/CDCP1 signaling axis in cancer cells blunts paracrine activation of cancer-associated fibroblasts (CAFs). In CRC–CAF co-culture systems (SW480 + CAF and HCT116 + CAF), treatment with the SMYD3 methyltransferase inhibitor BCI-121 or the Src kinase inhibitor SU6656 (a canonical CDCP1 effector) significantly reduced CAF transcripts of α-SMA, FAP, PDGFRβ, and S100A4 relative to DMSO (Figure 6A).

To corroborate pathway dependence, we perturbed the axis genetically in tumor cells. CAFs educated by shSMYD3 or shCDCP1 cancer cells displayed a marked reduction in protein levels of α-SMA, FAP, PDGFRβ and S100A4 compared with shNC controls (densitometry, mean ± SEM; *p* < 0.001; Figure 6B). Thus, blocking SMYD3 or CDCP1 is sufficient to switch off CAF activation programs induced by CRC cells.

We next evaluated therapeutic consequences in vivo using an experimental liver colonization model. Compared with vehicle, oxaliplatin (OXA) alone modestly reduced metastatic burden, whereas SMYD3 knockdown produced a greater decrease. Notably, combining sh-SMYD3 with OXA yielded the strongest antimetastatic effect: gross and histologic examinations revealed fewer/smaller lesions, with significant reductions in liver weight and metastatic area versus either monotherapy (Figure 6C–E). Immunohistochemistry demonstrated parallel stromal remodeling, with lower α-SMA and FAP positivity in the combination arm than in vehicle, OXA alone, or sh-SMYD3 alone (*p* < 0.001; Figure 6F,G). Survival analysis further showed prolonged survival with sh-SMYD3, and the longest survival with sh-SMYD3 plus OXA (log-rank test; Figure 6H).

In addition, to verify that the observed chemosensitization was not restricted to immunodeficient hosts, we reproduced the experiment in immune-competent C57BL/6 mice bearing syngeneic MC38 liver metastases. In this setting, sh-SMYD3 and oxaliplatin each reduced metastatic burden relative to Vehicle, with the combination yielding the greatest reduction across gross liver inspection, H&E-quantified metastatic area, and liver weight (*p* < 0.001; Supplementary Figure S1A–C). These findings confirm that SMYD3 suppression enhances oxaliplatin efficacy within an immune-competent context.

Collectively, these data show that therapeutic blockade of the SMYD3/CDCP1 axis disables tumor-promoting CAF activation, restrains hepatic metastatic outgrowth, and sensitizes CRC to oxaliplatin, supporting this axis as a tractable target to improve chemotherapy response.

## 4. Discussion

This study delineates a chromatin-encoded SMYD3–CDCP1 axis that links a nuclear epigenetic writer to a membrane signaling hub and promotes liver colonization in CRC. Convergent genetic, pharmacologic, and chromatin evidence indicates that promoter-proximal H3K4me3 at the CDCP1 promoter, correlating with SMYD3 activity, sustains CDCP1 transcription. Interrupting the axis at SMYD3, at CDCP1, or at proximal effectors attenuates EMT traits, deactivates CAFs, and increases oxaliplatin sensitivity in metastatic settings [22,23]. Collectively, these data nominate a therapeutically actionable pathway with biomarker potential.

Mechanistically, we position SMYD3 within a chromatin-centric paradigm of oncogenic regulation in CRC. By engaging the proximal CDCP1 promoter and promoting activating H3K4me3, SMYD3 drives transcriptional induction. Functional epistasis places CDCP1 downstream and indispensable for SMYD3-mediated migration, invasion, and metastasis. This hierarchy resolves the upstream control of CDCP1 in CRC and explains its prevalent overexpression and adverse prognostic association.

The cellular programs we observe are internally consistent with this axis. Sustained CDCP1 abundance enhances phosphorylation of Src (Y416) and PKCδ (Y311) without altering total kinase levels, maintaining cytoskeletal plasticity and an EMT-like state that lowers the threshold for invasion, vascular transit, and metastatic seeding [24,25,26]. Concordantly, transcriptome-level changes associated with SMYD3 activation enrich for pathways related to antigen processing and presentation, complement and coagulation cascades, phagosome formation, ECM–receptor interaction, and actin-cytoskeleton regulation [27,28,29]. Rather than implying immune activation per se, this pattern is most parsimoniously interpreted as a microenvironment configured for matrix remodeling, phagocytic turnover, and coordinated motility—features compatible with collective invasion [30]. In parallel, CDCP1-high tumor cells condition fibroblasts toward a myofibroblastic, pro-tumor phenotype (α-SMA, FAP, PDGFRβ, S100A4 upregulation), linking a tumor-intrinsic epigenetic program to stromal reprogramming. While these data establish pathway-level necessity for a fibroblast-activating secretome downstream of the SMYD3–CDCP1 axis, they do not resolve the specific mediator(s). We therefore interpret the CAF-activating effect as a paracrine program-level phenomenon; identifying causal cytokines or EV cargos will require targeted neutralization and receptor-level perturbation experiments.

Taken together, the ECM remodeling and CAF activation induced by the axis intersect with organ-level features that could favor hepatic colonization [31]. In particular, fenestrated sinusoidal endothelium and a readily activatable stellate-cell pool offer interfaces for adhesion, transendothelial passage, and early fibrotic priming [32]. While our study does not establish the sinusoidal architecture as necessary or sufficient for these effects, the concordance between axis-driven cues (ECM remodeling, CAF activation) and the known biology of hepatic stellate cells provides an anatomically plausible context for the pronounced liver colonization observed here [33,34,35].

The translational implications are two-fold. First, risk stratification: SMYD3 expression alone has prognostic value, but the combined assessment of SMYD3 and CDCP1 improves discrimination, suggesting that the axis—as a unit—better captures metastatic biology than either component in isolation. Second, therapeutic design: the pathway is targetable at multiple levels, including the enzymatic driver (SMYD3), the receptor hub (CDCP1), and proximal effectors (Src/PKCδ). The concordance between genetic and pharmacologic perturbations across these nodes provides a mechanistic benchmark for the development of more selective SMYD3 inhibitors and CDCP1-directed modalities (e.g., monoclonal antibodies, radioligands, antibody–drug conjugates) [36,37]. Notably, dampening the axis deactivates CAFs and reduces extracellular matrix (ECM) deposition (collagen I, fibronectin), leading to decreased matrix stiffness and interstitial pressure, improved vascular perfusion, and enhanced drug penetration. This microenvironmental normalization likely underlies the observed chemosensitization to oxaliplatin, a concept particularly relevant in liver-dominant metastatic CRC where desmoplasia and hypoperfusion restrict drug delivery [38,39,40].

Several features strengthen the proposed model. Cross-scale integration—from public transcriptomic datasets and institutional clinical cohorts to in vitro mechanistic assays and in vivo metastasis models—produced highly consistent results, underscoring the biological robustness of this axis. The chromatin evidence of SMYD3 occupancy and promoter-proximal H3K4me3 enrichment aligns with transcriptional upregulation of CDCP1 and downstream phenotypes such as enhanced migration, invasion, and liver colonization, thereby establishing a continuous mechanistic link from epigenetic regulation to metastatic behavior [41].

Beyond tumor-intrinsic signaling, our findings reveal a critical extension into the tumor–stroma interface. SMYD3-driven CDCP1 expression in cancer cells indirectly reprograms neighboring fibroblasts toward a myofibroblastic, pro-tumor phenotype, bridging nuclear epigenetic control with stromal activation [42,43]. This cross-compartment interaction suggests that tumor-intrinsic epigenetic dysregulation can remodel the stromal landscape to promote invasion and metastatic seeding, providing a conceptual framework that unifies tumor epigenetics and stromal biology within a single, experimentally tractable axis [44,45].

Several limitations merit consideration. Although our survival cohort was internally consistent and clinically well-annotated, its sample size was modest and single-center in nature. Validation in larger, multi-institutional cohorts with pre-specified cutoffs and standardized assays will be required to establish the clinical utility of the SMYD3/CDCP1 classifier [46]. Pharmacologic tools such as BCI-121 and SU6656 served as proof-of-concept reagents but exhibit suboptimal selectivity at higher concentrations, underscoring the need for next-generation inhibitors with improved potency and pharmacodynamic precision [47]. In addition, the present study did not assess the safety of SMYD3/CDCP1 inhibition in normal tissues. Future work will incorporate tumor-selective delivery strategies and systematic preclinical toxicology to evaluate potential on-target effects.

Moreover, the intrasplenic injection model recapitulates hepatic colonization and metastatic outgrowth but does not capture earlier steps of spontaneous dissemination, immune selection, or pre-metastatic niche formation [48]. The immune-competent C57BL/6–MC38 experiment mitigates the limitation of using nude mice by confirming that SMYD3 suppression enhances oxaliplatin efficacy in a T-cell–replete host. We did not profile immune subsets in this study; future work will address the immunological mechanism. Future work employing orthotopic and immunocompetent models will be essential to dissect how the SMYD3–CDCP1 axis influences early dissemination, immune evasion, and therapy resistance in a physiologically relevant context [49]. Integrating these models with spatial and single-cell transcriptomic profiling could clarify whether distinct CAF subsets (myCAF, iCAF, apCAF) or immune compartments preferentially associate with CDCP1-high tumor niches [50,51].

Finally, while our study establishes causality between SMYD3-mediated H3K4me3 and CDCP1 transcription, the precise mechanisms guiding SMYD3 recruitment remain undefined. Identifying the sequence motifs, cofactors, or chromatin accessibility patterns that anchor SMYD3 to the CDCP1 promoter will refine mechanistic understanding and may reveal additional regulatory partners. Such insights will inform the rational design of biomarker-driven therapeutic strategies that combine SMYD3 or CDCP1 inhibition with oxaliplatin, anti-fibrotic agents, or Src-pathway blockers in clinically relevant models.

## 5. Conclusions

In conclusion, our study delineates a chromatin-encoded SMYD3–CDCP1 axis that orchestrates epithelial–mesenchymal transition, cellular motility, and stromal activation to promote hepatic colonization in colorectal cancer. By functionally linking nuclear epigenetic programming to membrane signaling and stromal remodeling, this axis establishes a unified mechanistic framework underlying metastatic competence and therapeutic resistance. Disruption of SMYD3–CDCP1 signaling restores tumor–stroma homeostasis, enhances chemosensitivity, and highlights a promising avenue for biomarker-guided therapeutic intervention. Collectively, these findings identify the SMYD3/CDCP1 pair as both a prognostic biomarker set and a therapeutically exploitable vulnerability, underscoring its potential for further clinical translation in liver-dominant metastatic CRC.

## Data Availability

The datasets generated and analyzed during the current study are available from the corresponding author upon reasonable request. Publicly available datasets used in this study (e.g., GSE39582 and TCGA-COAD/READ) can be accessed through the GEO and TCGA databases.

## Supplementary Materials

The following supporting information can be downloaded at: https://www.mdpi.com/article/10.3390/biomedicines13112737/s1, Table S1. ShRNA sequences; Table S2. The primer sequences for qPCR were performed as follows; Table S3. Details of antibodies and reagents are as follows. Figure S1. (A) Representative livers, H&E whole-section images, and high-magnification fields from C57BL/6 mice bearing MC38 liver colonies following intrasplenic injection. White arrows mark metastatic nodules. In H&E panels, N denotes normal liver parenchyma and T denotes tumor. Dose and schedule of oxaliplatin as detailed in Methods. (B) Liver weight at endpoint. (C) Hepatic metastatic burden quantified as percentage metastatic area per section. Data are shown as mean ± SEM; each dot represents one mouse (n = 5). Statistical testing used one-way ANOVA with Tukey’s post-hoc comparisons across the four groups; *** *p* < 0.001.

## Abbreviations

AUC: area under the ROC curve; CI, confidence interval; COAD, colon adenocarcinoma; CRC, colorectal cancer; EMT, epithelial–mesenchymal transition; CAF(s), cancer-associated fibroblast(s); myCAF, myofibroblastic CAF; iCAF, inflammatory CAF; apCAF, antigen-presenting CAF; ECM, extracellular matrix; OS, overall survival; HR, hazard ratio; ROC, receiver operating characteristic; KEGG, Kyoto Encyclopedia of Genes and Genomes; TCGA, The Cancer Genome Atlas; GTEx, Genotype-Tissue Expression; SMYD3, SET and MYND domain-containing protein 3; CDCP1, CUB-domain containing protein 1.
