# Supplementary material for: SMYD3–CDCP1 Axis Drives EMT and CAF Activation in Colorectal Cancer and Is Targetable for Oxaliplatin Sensitization
Source: Biomedicines. 2025 Nov 9;13(11):2737. doi: 10.3390/biomedicines13112737 (PMC12650379; doi:10.3390/biomedicines13112737)
Supplement: Supplementary file 1 [file biomedicines-13-02737-s001.zip › biomedicines-3962244-supplementary.pdf]

Supporting Information

*Corresponding author

Zhaoxu Zheng: Department of Colorectal Surgery, National Cancer Center/National Clinical Research Center for Cancer/ Cancer Hospital, Chinese Academy of Medical Sciences and Peking Union Medical College, Beijing, China, Email: zzx_20003@126.com.

Supplemental Materials and Methods

Generation of SMYD3-overexpressing and CDCP1-silenced CRC cell lines

Human SMYD3 cDNA was cloned into pLenti-EF1α-Puro (empty vector as control), and CDCP1 knockdown employed pLKO.1-shRNA constructs. For dual selection, the SMYD3 vector conferred puromycin resistance and sh-CDCP1 conferred blasticidin resistance. CRC cells were transduced with LV-SMYD3 (MOI = 10 μg/mL polybrene), followed by puromycin selection (HCT116 2 μg/mL; SW480 1 μg/mL) for 5 days. SMYD3-OE pools were then infected with sh-CDCP1 lentivirus (MOI = 10) and selected with blasticidin (5 μg/mL, 5 days).

Primary CAFs used for co-culture

Primary CAFs were isolated from fresh colorectal cancer specimens of three unrelated, chemotherapy-naïve patients with informed consent. After enzymatic dissociation, fibroblasts were enriched by plastic adherence and differential trypsinization (two short digestion cycles). CAF identity was verified at passage 2 by qPCR showing upregulation of FAP and ACTA2 relative to epithelial-enriched fractions. Cells from passages 2–3 were used for co-culture experiments.

Syngeneic immune-competent validation (C57BL/6–MC38).
Six week-old male C57BL/6 mice were randomized prior to treatment. MC38 colorectal cancer cells (1×10^6 in 50 μL PBS) were injected into the spleen under anesthesia, followed by splenectomy to prevent local tumor growth. OXA was administered intraperitoneally at 5 mg/kg in 5% glucose, twice weekly for 3 weeks. Randomization was performed by an independent lab member, and outcome assessors were blinded to group allocation.

Supplementary Table S1. ShRNA sequences:

**Name**	**sequences (5'-3')**
ShNC	GCATTACGCGGATAACTCA
Sh-1-SMYD3	AGCCTGATTGAAGATTTGATT

Sh-2-SMYD3	GCTTCCCGATATCAACATCTA
Sh-CDCP1-F	CCGGCCTCAACTTCAATGTCTCCAACTCGAGTTGG AGACATTGAAGTTGAGGTTTTTG
Sh-CDCP1-R	AATTCAAAAACCTCAACTTCAATGTCTCCAACTCG AGTTGGAGACATTGAAGTTGAGG

Supplementary Table S2. The primer sequences for qPCR were performed as follows：

Name	Forward primer (5'-3')	Reverse primer (5'-3')
SMYD3	GAAAAGTTCGCAACCGCCAA	GCATCAGCTTTTCCTTCCCGA
CDCP1	CCCCAAGGACTGTGGACTTG	ACAGCAAATGATGAGCCCGA
CORIN	CCTCCGGTTCCTATTGCTGG	TGTTCATACAGGCACCAACAT
ADAMTS15	GGCCCACCCCTATTGTATGG	CTCCACACAGGTCCTTGGAT
SEMA3E	TGCCTTCCCTTAGATGAACAGAG	ACTCGGCCAGTGTATCTCTT
CSPG5	GCCATAGTTCTGCTGAGAAACA	GCCTCACGCGCCAAATG
ACTA2	TATCCCCGGGACTAAGACGG	CACCATCACCCCCTGATGTC
FAP	TCTGGAAAAATGAAGACTTGGGT	AGGGCGTAAGACAATGCACA
PDGFRβ	CCATCAGCAGCAAGGCGA	AGCAGGTCAGAACGAAGGTG
S100A4	GCTTCTTCTTTCTTGGTTTGATCCT	ACTTGTCACCCTCTTTGCCC
P1	ACCGGCTTTAAAGTCCAACAG	GAGGAGACAAAGGCCATTATG
P2	ACCATCCGCAATCTCTGCCTG	TTTCCAAATCCTCCCAGCCGC

Supplementary Table S3. Details of antibodies and reagents are as follows:

Reagent or Resource	Source	Identifier
**Antibodies**		
GAPDH	Proteintech	Cat. No. 10494-1-AP
SMYD3	Abcam	Cat. No. ab228015
H3K4me3	CellSignalingTechnology	Cat. No. 9751
α-SMA	CellSignalingTechnology	Cat. No. 19245
E-cadhein	CellSignalingTechnology	Cat. No. 3195
ZEB1	CellSignalingTechnology	Cat. No. 3396
Snail	CellSignalingTechnology	Cat. No. 4719
CDCP1	CellSignalingTechnology	Cat. No. 4115

p-Src(Y416)	Sigma	Cat. No. SAB5700368
SRC	CellSignalingTechnology	Cat. No. 2180
p-PKCδ(Y311)	Abcam	Cat. No. EPR2609Y
PKCδ	CellSignalingTechnology	Cat. No. 2058
FAP	CellSignalingTechnology	Cat. No. 52818
PDGFRβ	CellSignalingTechnology	Cat. No. 3169
S100A4	Proteintech	Cat. No. 16105-1-AP
IgG	CellSignalingTechnology	Cat. No. 2729

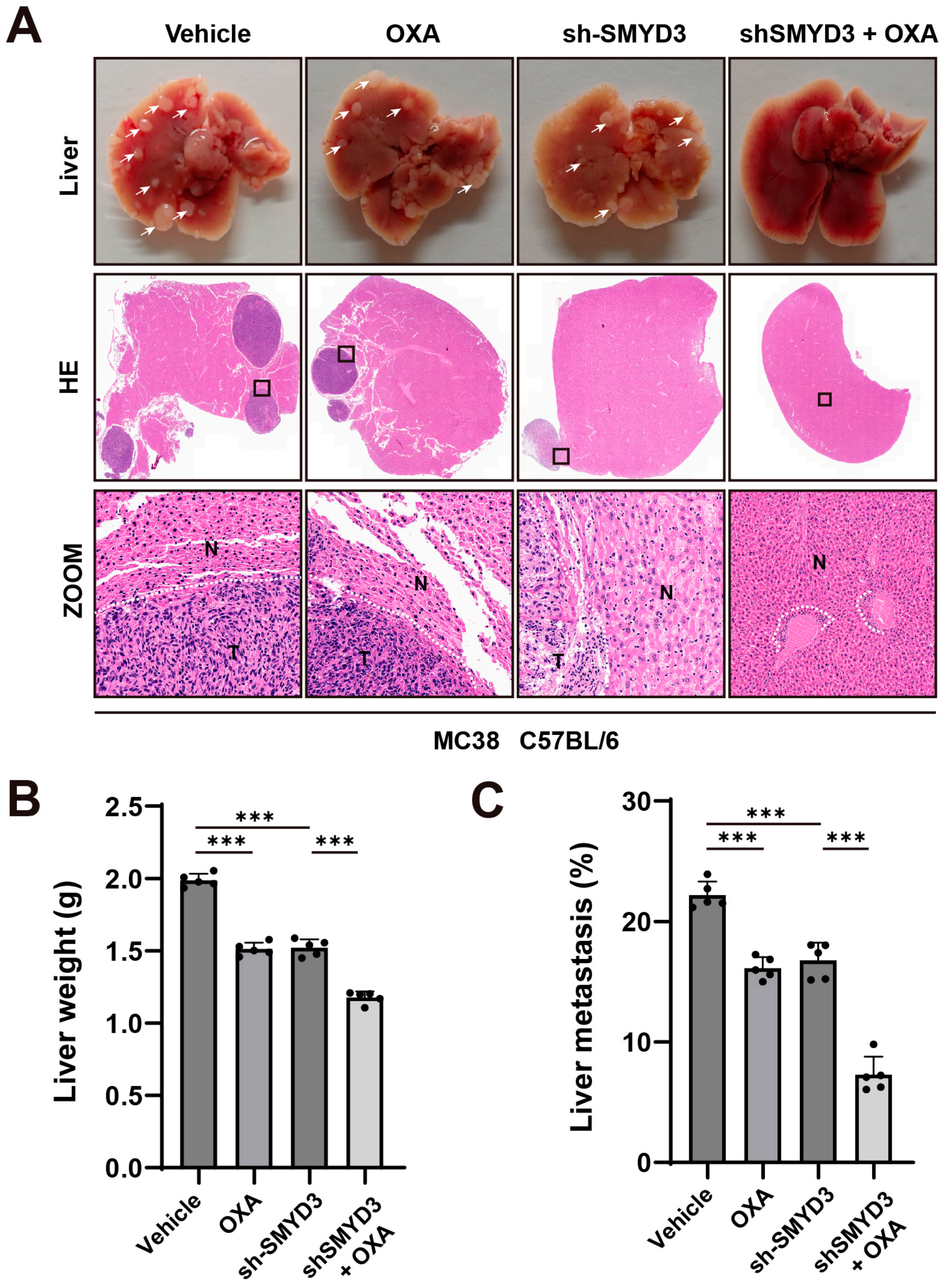

Supplementary Figure S1

(A) Representative livers, H&E whole-section images, and high-magnification fields from C57BL/6 mice bearing MC38 liver colonies following intrasplenic injection. White arrows mark metastatic nodules. In H&E panels, N denotes normal liver parenchyma and T denotes tumor. Dose and schedule of oxaliplatin as detailed in Methods. (B) Liver weight at endpoint. (C) Hepatic metastatic burden quantified as percentage metastatic area per section.

Data are shown as mean ± SEM; each dot represents one mouse (n=5). Statistical testing used one-way ANOVA with Tukey's post-hoc comparisons across the four groups; ***$P < 0.001$.
